# Recent insights on direct democracy: Arguments, drivers, effects and conditions

**DOI:** 10.12688/openreseurope.20444.1

**Published:** 2025-06-27

**Authors:** Caner Şimşek, João Limão, Inês Campos, Doris Fuchs, Bernd Schlipphak

**Affiliations:** 1 Institute of Political Science, University of Münster, Münster, Germany; 2 Centre for Ecology, Evolution and Environmental Changes, Faculty of Sciences of the University of Lisbon, Lisbon, Portugal; 3 Research Institute for Sustainability (RIFS) at GFZ, Potsdam, Germany

**Keywords:** direct democracy; review; referendum, ballot initiatives

## Abstract

Rooted in history, with origins tracing back to Athenian democracy in the 5th century BCE, direct democracy has gained renewed attention as a potential solution to the challenges of representative democracy. It is often seen as a means to reduce democratic deficits, enhance citizen participation, and legitimize political decisions. This study reviews recent literature on direct democracy, analyzing 46 articles published between 2016 and 2023 to map key themes and debates. While findings highlight its potential to strengthen participation, empower citizens, and align policies with public preferences, they also emphasize significant risks, including the marginalization of vulnerable groups and susceptibility to elite manipulation.

## Plain Language Summary

Direct democracy, where citizens vote directly on laws and policies, has been around since ancient Athens and is now being looked at again as a way to improve how modern democracies work. Many people believe it can help fix problems like low trust in politicians and lack of citizen involvement. This study looked at 46 recent research papers published between 2016 and 2023 to understand what experts are saying about direct democracy today. The research shows that while direct democracy can increase participation and make government decisions more in line with what people want, it also comes with risks. These include the possibility of powerful groups taking advantage of the system and some groups of people being left out or harmed by the process.

## Introduction

Broad public support for democracy as a political system coexists with growing skepticism about its quality. Modern liberal democracies have been questioned and accused of distorting some of their essence, and citizens express their cynicism and dissatisfaction through lower participation and voting for disruption. Democratic mechanisms of accountability have been challenged by globalisation and the market ( Della Porta, 2013), while democracies increasingly face persistent threats such as fragmentation and polarization ( McCoy *et al.*, 2018; Orhan, 2022; Sunstein, 2002), populist movements ( Gerber, 2011; Mudde, 2004; Mudde & Kaltwasser, 2017; Pauwels, 2014), and the broader trend of democratic backsliding ( Bermeo, 2016; Wolkenstein, 2023). In response to these challenges, direct democracy has gained attention as a potential remedy, emerging as a potential alternative or solution to some of the difficulties ( Bernhard, 2024).

The goal of this review article is to take a snapshot of the current literature and its assessment of direct democracy’s potential as a solution to the aforementioned problems. By examining 46 recent articles, we aim to map the topics being explored under the umbrella of direct democracy and highlight the key debates that animate this growing field of research. Direct democracy, as both a political mechanism and an area of study, has seen significant scholarly attention due to its perceived potential to address democratic deficits, enhance citizen participation, and produce more legitimate policy outcomes. However, it remains a contested terrain, with diverse perspectives on its drivers, effects, and conditions for success. In this review, we focus on synthesizing insights around four central questions: What drives the adoption of direct democracy? Under what conditions does it succeed? What are its effects? And how do these dynamics play out and intersect in different political and institutional contexts?

To address these questions, we used a sample of articles published between 2016 and 2023, as the seminal article of Leeman and Wasserfallen (2016) seems to have started a new wave of research on the topic. The sample was constructed by searching articles that fulfilled the following criteria: First, they needed to be published between 01.01.2016 and 30.10.2023 (the time the search ended). Second, they needed to include the term “direct democracy” in the title. As a result, there may be additional published research addressing the topic, but we are confident that the work in which direct democracy is the most salient concept has been included in the sample. The initial sample was constructed through a comprehensive search conducted by a student assistant, who began with Google Scholar and then expanded the search to include the Scopus database as well as the University of Münster’s local library catalogue. This resulted in a dataset of 46 articles published in journals relevant to the Political Science community. After careful reading, we found that three of these articles were irrelevant for the purpose of this review and did not fit its scope. Therefore, we reviewed 43 articles. We present a thematic review of the literature rather than a systematic review and therefore do not claim to exhaustively or comprehensively cover all relevant studies.

We distinguish between effects, conditions, and structural versus case-specific arguments, considering each along short-term and long-term dimensions where appropriate. By doing so, we aim to uncover both the immediate, context-dependent factors and the broader, more enduring dynamics that shape the adoption, operation, and outcomes of direct democracy. This distinction is crucial for several reasons. First, it reflects the multifaceted nature of direct democracy, which operates both as an immediate response to political pressures and as a long-term institutional feature. Short-term dynamics often capture the situational drivers or outcomes of direct democracy, such as a referendum triggered by a political crisis or the immediate populist boost following a successful initiative by corresponding actors. In contrast, long-term dynamics focus on broader, systemic influences like political culture, institutional traditions, and socio-economic development, which are likely to shape the adoption and effectiveness of direct democracy over time.

We conclude that while direct democracy can potentially be beneficial, it is not without risks. It offers potential benefits such as enhanced citizen participation, greater legitimacy, and policy alignment with public preferences. While early research emphasized its educative and egalitarian potential, recent studies highlight significant challenges, including overlaps with populism, risks to marginalized groups, and concerns about citizens' competence in complex policymaking. Additionally, direct democracy is vulnerable to manipulation by political elites, particularly in illiberal contexts, where referendums can be exploited to suppress opposition, entrench power and undermine minority rights. Without institutional safeguards, these risks can erode democratic principles, destabilize governance and exacerbate societal divisions.

## Discussion

### Deficits and cultural shifts

The introduction of direct democracy is often driven by complex and long-term factors that mirror shifts in political culture and public values. One of the arguments for the introduction of direct democracy is the desire to assert citizen control over the administrative state. Kogan, Lavertu, and Peskowitz (2017) argue that citizens seek greater influence over administrative processes to address perceived inefficacy, particularly in managing public resources. In particular, perceptions of democratic deficits serve as powerful drivers for the introduction of direct democratic mechanisms. When citizens feel that representative institutions fail to reflect their preferences or uphold democratic values, they may advocate for direct democracy as a corrective measure ( Leemann & Wasserfallen, 2016). Direct democracy, with its emphasis on direct citizen involvement in decision-making, addresses many of the grievances tied to the democratic deficit. By allowing citizens to participate more actively in the political process through referendums and initiatives, direct democracy offers a way to counteract declining trust in elites, foster greater political engagement, and increase transparency in governance. Vatter, Rousselot, and Milic (2019) argue that direct democracy can empower citizens and promote more responsive governance by aligning policy outcomes more closely with voter preferences, thereby helping to mitigate the democratic deficit. Their literature review highlights studies suggesting that direct democratic mechanisms may also constrain public spending and enhance the efficiency of service delivery.

Earlier works also noted how direct democracy functions as a corrective mechanism for political discontent. For instance, Dalton, Burklin, and Drummond (2001) argue that as citizens grow more dissatisfied with traditional political institutions, they seek out alternative methods to express their political preferences. Moreover, as representative institutions are seen as increasingly detached from the electorate, direct democratic tools are viewed as a means of restoring accountability and ensuring that political outcomes better align with public preferences.

Political cynicism and the rise of conspiracy theories also play a significant role in fostering support for direct democracy. Pantazi, Papaioannou, and Prooijen (2022) examine what they describe to be the hidden link between belief in conspiracy theories and a preference for direct democracy. Their findings indicate that conspiracy theorists, who tend to distrust representative democracy, often view direct democracy as a more effective system for expressing their political agency. This phenomenon is mediated by political cynicism and feelings of powerlessness. These findings collectively yield a critical insight: as citizens lose faith in traditional representative institutions, they increasingly turn to direct democracy as an alternative that promises more direct control over political outcomes.

The cultural and political transformations associated with postmaterialism have also led to an increase in the adoption of direct democracy. As societies have moved away from concerns over material well-being to emphasize issues such as individual autonomy, environmental sustainability, and human rights, political parties have been compelled to adjust. Qvortrup (2017) argues that postmaterialist values have pressured political actors to handle contentious and high-stakes issues through referendums. In this context, parties cannot afford to alienate their voters by taking unilateral positions on controversial topics; instead, they delegate these issues to the electorate itself. Qvortrup’s analysis challenges the common narrative that referendums are primarily tools of populists and autocratic leaders. Rather than being exploited by authoritarian regimes, the referendum has been more frequently employed in well-functioning democracies, such as Switzerland, as a mechanism for enhancing democratic legitimacy and involving citizens in significant political decisions.

In light of these arguments, the push toward direct democracy can be seen as a response to shifting societal demands. The post-material revolution has led to the emergence of new political cleavages, making it difficult for traditional representative democracies to address diverse and often conflicting demands. Direct democratic mechanisms offer a means of navigating these divides by giving citizens a more direct say in policymaking. As citizens place increasing value on participation and self-expression ( Inglehart, 1997), the institutional design of democracy has adapted to accommodate these preferences.

Importantly, the introduction of direct democracy does not occur in a vacuum. Historical legacies, institutional arrangements, and the broader political context shape how and why direct democracy emerges. For instance, the seminal work of Altman (2011) notes that countries with long-standing traditions of local governance and civic participation are more likely to embrace direct democratic reforms. Similarly, the presence of strong constitutional constraints, as highlighted by Qvortrup (2017), ensures that referendums and initiatives are used as democratic safeguards rather than populist tools.

In summary, direct democracy offers a mechanism for addressing these evolving demands, providing citizens with a more direct role in shaping political outcomes. As the literature demonstrates, the adoption of direct democratic practices reflects both a response to political discontent and a broader evolution in democratic governance.

### Weapon of the weak or tool of the elite?

One of the most common short-term drivers for introducing direct democracy is the rise of citizen initiatives, protests, and awareness campaigns, which act as catalysts for political change. According to Tosun, Béland, and Papadopoulos (2022), citizen initiatives, such as the European Citizens’ Initiatives (ECIs), have proven to be effective tools for introducing incremental policy changes in the European Union. While not legally binding, these initiatives exert indirect influence on political systems, forcing politicians and institutions to address public demands. The authors highlight how ECIs can be transformative over time, serving as building blocks for larger policy reforms.

The rise of direct democracy as a tool for addressing public dissatisfaction has also been evident in protest movements such as the Yellow Vests in France. Van Outryve (2023) documents how the movement criticized the representative government and called for more direct democratic mechanisms. The Yellow Vests proposed the establishment of local assemblies to bypass the limitations of representative democracy and grant more power to citizens. This experiment in the municipality of Commercy exemplifies how protest movements, driven by short-term dissatisfaction with existing governance structures, can push for the introduction of direct democracy as a solution to address power imbalances and perceived governance failures. In this context, direct democracy is seen as a means to give power back to the people, reflecting a grassroots effort to rectify the shortcomings of representative systems.

Direct democracy is not only driven by bottom-up initiatives but is also frequently employed as a top-down political strategy by elites to achieve specific political objectives. Gherghina (2019) discusses how political parties in Romania have used referendums to legitimize their positions, boost their popularity, and outmaneuver political opponents. In post-communist Romania, seven national-level referendums were primarily employed as tools for electoral strategy rather than genuine mechanisms for gauging public opinion. In these cases, political actors used direct democracy to gain legitimacy or distract the public from controversial issues. This instrumental use of referendums reveals how elites can manipulate direct democracy for short-term political gains rather than addressing broader democratic deficits.

Similarly, Pállinger (2019) highlights how Hungarian political elites have used referendums to legitimize controversial legislative projects and preempt potential election issues. In Hungary's increasingly illiberal political environment, ruling elites have called referendums to push through legislative agendas, often in the face of opposition from minority political actors. This illustrates another short-term driver of direct democracy: elites may strategically employ referendums to solidify their political dominance, limit opposition, and avoid future electoral challenges. Thus, direct democracy can be hijacked for short-term political manipulation, serving elite interests rather than those of the broader public. Moreover, Cortés (2019) emphasizes the growing use of referendums and initiatives to decide policy issues worldwide. In cases where governments face institutional resistance to their legislative agendas, direct democratic tools provide a means to bypass opposition and quickly implement policy changes. The speed and decisiveness that direct democracy makes it an appealing solution for political actors aiming to push through their policies in the short term.

Direct democracy frequently emerges in response to immediate political needs and short-term grievances. Whether used as a tool for policy change, as seen with European Citizens’ Initiatives, or as a political weapon by elites, direct democracy provides a mechanism to address pressing political, economic, and social issues. Further research is needed to explore how direct democracy can be better harnessed to serve democratic ideals without succumbing to elite capture or strategic manipulation.

### Transformative changes, impacts and inconclusive effects

The literature discusses the potential effects of direct democracy across a wide range of areas, including politics, economics, education, taxation and representation. These effects may arise directly through the policies enacted via these instruments, or indirectly through their influence on participants and institutions. Based on a set of popular consultations that took place in Colombian municipalities regarding citizens' views on extractive industries in their territories, Acosta García ( Acosta García, 2022) argues that popular consultations, as spaces of experimental autonomous activity have the potential to engender alternatives to extractivist development. Economou, Kyriazis and Metaxas (2017) compare three historical cases of direct democracy (ancient Athens, modern California and modern Greece) to examine institutional settings, deliberative processes, and outcomes. In Athens, direct democracy promoted the public good; in California, it primarily aimed to increase disposable income; while in modern Greece, the 2015 referendum had no practical effect, as its result was not implemented.

One major area of debate concerns the relationship between direct democracy and attitudes towards politics. In an analysis on the German Bundesländer, ( Ackermann *et al.*, 2023) emphasize that citizens in contexts with direct democratic mechanisms do not exhibit more populist attitudes nor greater support for the political system, confirming the same result of previous research, which also did not detect such a correlation ( Ladner & Fiechter, 2012; Mendelsohn & Cutler, 2000; Smith & Tolbert, 2004). Also, Gherghina and Pilet (2021) identified limitations in the studies that associate populist attitudes with direct democracy, demonstrating that these analyses have theoretical, conceptual and methodological weaknesses that do not support this correlation. Using a structural equation model, Chang (2023) shows that political efficacy mediates the relationship between support for direct democracy and party affiliation: citizens with high internal and external efficacy who value direct democracy are more likely to be affiliated with a political party. In contrast, the simple availability of direct democratic mechanisms does not appear to influence party affiliation directly.

Previous studies suggest that overly frequent use of popular votes can erode citizens’ trust in political institutions, as people generally prefer effective systems without bearing the burden of continuous decision-making themselves ( Hibbing & Theiss-Morse, 2002). However, Freitag and Ackermann (2016) challenge the universality of this claim, arguing that responses to direct democracy vary across personality types. Using hierarchical data from 26 Swiss cantons, they demonstrate that while direct democracy can shape institutional trust, the direction and strength of this effect are mediated by individual personality traits. Specifically, characteristics such as openness to experience, agreeableness, conscientiousness, extraversion, and neuroticism influence how citizens perceive and react to popular votes, suggesting that some individuals are more responsive to democratic stimuli than others. In a broader exploratory analysis of 38 European countries, Gherghina (2017) identifies a positive association between the presence of direct democracy at the local and national levels and the perceived legitimacy of political regimes. These findings suggest that direct democratic instruments may contribute to enhancing governmental legitimacy.

The potential effects on education and knowledge are also inconclusive at best. ( Barth *et al.*, 2020) question the idea that direct democracy increases attention to politics, since one of the conditions for educational effects is voters' familiarity with initiatives and referendums. When asked about their knowledge on concrete policy aspects and measures, many citizens reveal a much lower level than they claimed. Public awareness of the measures is lower than previous studies suggest, demonstrating that the educational effect of direct democracy is implausible. Others, such as Adam and colleagues (2018), who studied the Greek, Scottish and Catalan referendums, found no correlation between direct democracy and increased interest in politics and political education, just as they considered the relationship between direct democracy and populism to be inconclusive.

Using data from the European Social Survey, Kern and Hooghe (2018) show that more educated citizens benefit from the greater abundance of participatory tools, while citizens with fewer educational resources show a fatigue that leads them to participate less , accentuating the gap in political influence between different segments of the population. Another correlation that emerges as inconclusive is between direct democracy and greater equality. However, Geißel and colleagues (2019) clarify that there are more pro-equality bills than those that have the opposite effect.

In terms of voter turnout, both in local and national elections, a study carried out in Czechia revealed a strong direct positive correlation between direct democracy campaigns and increased voting levels ( Dvořák *et al.*, 2017). This positive correlation is in line with previous results ( Childers & Binder, 2012; Dyck & Seabrook, 2010). However, contrasting evidence comes from a cross-national panel study covering democracies from 1980 to 2005, which found that direct democracy mechanisms can take over functions traditionally performed by representative institutions, potentially leading to a decline in electoral participation. Despite advocates' expectations of complementarity, the study observed a drop in turnout where direct democracy tools were institutionalized ( Peters, 2016).

Several studies show that direct democracy can have immediate effects on policies and society. Tosun, Béland and Papadopoulos (2022) point out that the introduction of direct democracy institutions can have direct impacts that lead to transformations in society, examples of which are the national sovereignty referendums and the Brexit vote. Direct democracy, especially in the form of constitutionally incorporated referendums, has been suggested as a possible solution to correct systemic flaws in a representative democratic system. However, an empirical study by But *et al.* (2023) examining the relationship between the use of direct democratic instruments and countries’ Democracy Index Scores found that while there is a positive correlation globally, this relationship does not hold within the sample of EU countries.

Kersting and Regalia ( Kersting & Regalia, 2022) applied the Direct Democracy Integrity Index to examine constitutional referendums in Turkey (2017) and Italy (2020), investigating their roles in shaping democratic governance. Using a most different systems design, they compared a modern autocracy (Turkey) and a consolidated democracy (Italy), both of which frequently use nationwide constitutional referendums. Their analysis shows that while the Turkish referendum contributed to majoritarianism by consolidating presidential power, the Italian case provided an opportunity for minorities and regional actors to express dissent.

Leeman and Wasserfallen ( Leemann & Wasserfallen, 2016) draw on the literature and empirical research based on data from 10 Swiss cantons to affirm that direct democracy initiatives have a positive effect on the congruence of policies with voters' expectations. While some previous studies have also identified such a relationship ( Matsusaka, 2004; Matsusaka, 2010), others did not find evidence of this correlation ( Lascher *et al.*, 1996; Lax & Phillips, 2012; Tausanovitch & Warshaw, 2014). The authors explain this difference by showing that DD does not have a single, constant effect on congruence, verifying that the increase in congruence occurs more frequently when the preferences of the people and the elite diverge.

Instruments of direct democracy, such as initiatives and referendums, are argued to positively influence how members of parliament (MPs) perceive public opinion on various policy proposals. A survey conducted among national parliamentarians and citizens found that MPs more accurately assess proposals that are subject to a direct democratic vote, suggesting that popular votes serve as a valuable source of information for aligning with citizen preferences ( Helfer *et al.*, 2021). Previous research has shown that the mere possibility of a referendum can induce MPs to get closer to voters ( Hajnal *et al.*, 2002; Matsusaka & McCarty, 2001).

Based on the premise that direct democracy may enhance gender equality by creating more inclusive spaces for political engagement and by affirming women's political competence, Kim ( Kim, 2019) investigated the effect of direct democracy on gender equality by using historical data from Sweden. Analyzing the period between 1921 and 1940 that followed the introduction of universal suffrage, the study compared female electoral participation across municipalities with and without direct democracy mechanisms. The findings indicate that the gender gap in voter turnout was smaller in municipalities that employed direct democracy tools than in those relying solely on representative mechanisms.

To sum up, direct democracy can have transformative effects on society, contributing to the generation of tangible democratic alternatives by allowing citizens to present proposals in some cases, and by increasing the congruence of policies with citizens' expectations, being a relevant source of information for MPs.

### Prerequisites for successful operation

The effectiveness of direct democracy depends significantly on the conditions under which it is implemented. Although recent literature on this topic remains relatively limited, several studies have addressed the contextual factors that shape the successful functioning of direct democratic instruments. As with all democratic processes, sustained citizen support is a crucial prerequisite. Without broad-based public engagement and trust, the legitimacy and practical impact of direct democracy may be undermined, regardless of the institutional design. Adinolfi (2018) using multilevel analysis in the Portuguese context, found widespread support for democracy in general, as well as for specific mechanisms like direct democracy. At the legislative level, various mechanisms are employed to regulate and safeguard the implementation of direct democracy initiatives. In Romania, for example, Gherghina (2019) highlights how minimum quorum requirements are used to validate referendum outcomes, serving as a legal constraint on their effectiveness.

To assess the conditions under which direct democracy institutions can develop Altman (2017) created the Direct Democracy Practice Potential Index. This index was applied to 200 policies across various countries between 1900 and 2014, evaluating four types of mechanisms: popular initiatives, popular referendums, obligatory referendums, and authorities’ plebiscites. The index captures the ease of initiating, implementing and approving direct democratic measures, as well as the significance of the resulting vote in terms of legal bindingness and practical enforcement.

The dissemination of relevant information during referendum campaigns plays a crucial role in shaping individuals’ positions and is generally valued by citizens. However, the arguments circulated in these campaigns are often closely aligned with party political orientations, which in turn influence voters’ motivations both directly and indirectly. Citizens do engage with policy information, but primarily when it aligns with their existing partisan views ( Colombo & Kriesi, 2017). In response to this challenge, Merli (2020) advocates for the introduction of additional safeguards, such as the provision of high-quality, independent information that focuses on the content and likely effects of proposed measures. She also emphasizes the importance of allowing sufficient time and opportunity for deliberation, enabling citizens to form more informed and reasoned opinions and mitigating the risk of knowledge deficits.

More broadly, analyzing the conditions for the emergence and implementation of direct democracy requires careful contextualization. Historical, cultural, social, and legislative factors can all condition the development and functioning of direct democratic practices. For example, the availability of accurate information, opportunities for effective deliberation, and an understanding of the implications of policy choices are all critical enabling conditions.

### Systemic strengths and weaknesses of direct democracy

While the early literature emphasized that direct democracy can strengthen citizens' capabilities and knowledge, contributing to a higher quality of democracy ( Kriesi, 2012), more recent studies, on the other hand, are skeptical about this educational aspect. Ackermann *et al.* (2023), for example, highlight that while direct democracy offers citizens a more direct voice in decision-making, potentially addressing democratic malaise, it also bears conceptual similarities with populist appeals, raising concerns about its educative versus populist potential. Similarly, Mendez and Mendez (2017) note that, although they are optimistic about the ECI, citizens are not competent enough to understand the decision making in the EU. They further warn that how direct democracy may delegitimize the EU's complex policymaking structures.

Legitimacy is another recurrent theme and the literature addresses how citizens perceive the fairness of laws passed through direct democracy versus representative democracy. Ladam (2020) finds that citizens tend to view outcomes from ballot initiatives as fairer than those passed through legislative processes, suggesting that the participatory nature of direct democracy fosters a perception of fairness (although it does not change their evaluation of outcomes). Mendez and Mendez (2017) also suggest that referendums grant a high degree of legitimacy to a political decision in general but much also depend on different types of EU referendum. Similarly, Towfigh *et al.* (2016) argue that for issues that are deemed important for voters, direct democracy generates greater acceptance even when those voters who do not agree with the collectively chosen outcome.

Direct democracy can also serve as a corrective tool, pushing the policymakers to pay more attention to public preferences ( Leemann & Wasserfallen, 2016). Tosun *et al*. (2022) argue that instruments of direct democracy, such as the ECI, can foster incremental yet transformative policy changes. They contend that direct democracy can discipline policymakers, forcing them to consider the preferences of citizens to avoid the risk of veto. Prato and Strulovici (2017) similarly highlight the role of direct democracy in aligning policies with the electorate's preferences, arguing that it typically improves congruence between policy outcomes and voter desires. However, they also caution that this benefit may come at the expense of political institutions, as direct democracy can undermine the role and incentives of elected officials, leading to long-term negative effects on governance. Walter (2019) extends this analysis to welfare state expansion, showing that direct democracy can either constrain or expand social expenditure depending on the political environment. In settings with few governing parties, direct democratic instruments can expand welfare provisions, while in settings with many governing parties, they tend to constrain social spending.

A separate line of inquiry focuses on the egalitarian potential of direct democracy. Asimakopoulos (2016) presents an idealistic view, advocating for direct democracy as a means to promote substantive equality. He argues that traditional electoral systems are inherently fraudulent and that only direct democracy can provide genuine equality. Krämling *et al.* (2023) adopt a similar stance, through a comparative study of direct voting practices in countries of the Global South (including the Caribbean, Latin America, Africa, Asia, and Oceania) between 1990 and 2015. Their analysis reveals that nearly half of the votes concerned socio-economic issues or legal and political equality, with most aiming to enhance equality and showing a considerable likelihood of success. The findings suggest that direct democracy is not inherently a threat to equality and that direct democratic mechanisms tend to produce more pro-equality outputs than expected. The study observed consistent pro-equality outcomes across diverse political cultures. Contrary to fears that direct democracy might exacerbate inequality. Similarly, Kim (2019) argues that direct democracy can foster political inclusion, particularly for women.

However, this optimistic view is not without its critics. Some studies show that direct democracy can also systematically disadvantage marginalized groups, as evidenced by more negative outcomes for racial and ethnic minorities in direct democratic processes ( Hainmueller & Hangartner, 2019). Thus, while direct democracy holds promise for promoting equality, its implementation may still produce unequal outcomes for vulnerable groups. Merli (2020) echoes this warning by arguing that direct democratic instruments can be used to curtail minority rights, thereby reinforcing illiberal democracies.

The relationship between direct democracy and deliberation has long been debated, with deliberative democracy theorists traditionally viewing direct democratic mechanisms as incompatible with deliberative ideals. Critics argue that these instruments promote a purely procedural form of democracy, lacking the substantive discourse central to deliberation. However, ( el-Wakil, 2017) challenges this assumption, suggesting that optional referendums align with Joshua Cohen’s definition of substantive democracy by incentivizing political actors to provide acceptable justifications for their positions. A further example of deliberation within direct democratic settings is the Landsgemeinde, an annual open-air citizens’ assembly in Switzerland. An analysis by Gerber and Mueller (2018) of 500 speeches delivered between 2000 and 2012 demonstrates that open citizen participation in such forums does not undermine democratic functioning but instead illustrates the capacity of citizens to actively engage in public discourse. Expanding on this debate, ( el-Wakil & McKay, 2020)) argue that the perceived tension between direct and representative democracy is largely unfounded. They advocate moving away from a narrow "Direct Democracy Approach" toward a broader "Democratic Systems Approach" that integrates various forms of decision-making such as elections, town hall meetings, and mini-publics within a unified democratic framework.

### Contextual critiques of direct democracy

Several scholars highlight how direct democracy, while theoretically a channel for public will, can be manipulated by political elites and incumbents to secure partisan advantage, restrict political competition, or promote illiberal agendas. For instance, Gherghina (2019) shows that in Romania, referendums were primarily used by political elites for electoral gains or legitimacy rather than for genuine democratic deliberation. Two out of the seven referendums were directly aimed at weakening political opponents, illustrating how the ruling elites monopolized the initiative power, with bottom-up referendums virtually impossible. Similarly, Merli (2020) emphasizes the illiberal potential of direct democracy, arguing that instruments such as referendums lack the parliamentary filters that normally protect minority rights. This absence of procedural checks can deepen societal cleavages, promote hate speech, and support authoritarian regimes.

Pállinger (2019) further expands on this in the Hungarian context, where direct democracy, under an increasingly illiberal regime, has been used to solidify political dominance. Despite Hungary's history of nation-wide referendums, direct democracy remains unclear in its democratic purpose, often serving to reinforce majoritarianism rather than fostering deliberative or participatory forms of governance. Adding a broader European dimension, Mendez and Mendez (2017) caution against the extra-territorial impacts of referendums in the EU. Political elites frequently exploit referendums for partisan advantage, especially in treaty revisions. This misuse can destabilize EU governance, eroding the legitimacy of the referendum as a democratic tool. Collectively these studies underscore the vulnerability of direct democracy to political manipulation, particularly in illiberal contexts, where ruling elites exploit referendums to undermine political competitors, entrench power, and push illiberal agendas.

Secondly, scholars have discussed how direct democracy can disrupt good governance. Kogan *et al*. (2017) examine the administrative consequences of failed referendums in Ohio school districts. They find that direct democracy can force administrators into protracted bargaining processes with voters, which can disrupt public service delivery. The key takeaway here is that direct democracy can introduce short-term transaction costs in governance, potentially with long-lasting effects. Similarly, Prato and Strulovici (2017) consider how direct democracy impacts politicians' behavior and incentives. They argue that increasing voters’ power to amend policy through referendums weakens politicians’ incentives to invest in expertise or perform effectively. The theory suggests that when voters can directly amend policies, politicians become less motivated to acquire competence, leading to poorer governance outcomes. Thus, direct democracy, while empowering voters, may undermine the very political competence and accountability it seeks to enhance.

## Conclusion

This article aimed to offer a review of the scholarly literature on direct democracy, not a direct assessment of empirical evidence. Accordingly, we do not make claims about what direct democracy does. Instead, we synthesize what existing research has found by higlighting prevailing arguments, debates and patterns across the literature. Since this literature review focuses on articles published in English between 2016 and 2023, this defined scope allows for a concentrated analysis of recent scholarly developments. However, it also means that research published in other languages or outside this timeframe is not included. Future reviews could expand this scope both geographically and temporally to incorporate a broader range of perspectives and potentially capture insights from different regions and historical contexts that may further enrich the understanding of direct democracy.

Direct democracy can potentially enhance democratic participation, strengthen citizen capabilities, and align policy outcomes with public preferences. Its educative benefits fostering a more informed and engaged citizenry notwithstanding, more recent studies highlight significant challenges, including its conceptual overlap with populism and concerns about citizens' competence in complex policymaking contexts, particularly in settings like the European Union. Direct democracy is also seen as a tool for enhancing legitimacy, with evidence suggesting that ballot initiatives are perceived as fairer and more participatory than legislative decisions. At the same time, instruments such as referendums can serve as corrective mechanisms, pushing policymakers to align more closely with public preferences and fostering incremental but meaningful policy changes.

Nevertheless, direct democracy is not without its risks. It can systematically disadvantage marginalized groups, with studies showing negative impacts on racial and ethnic minorities and potential threats to minority rights. While some scholars highlight its egalitarian potential, particularly in promoting political inclusion and pro-equality outputs, others caution that these benefits may come at the expense of institutional stability and long-term governance.

Direct democracy is highly vulnerable to manipulation by political elites, particularly in illiberal contexts. Instead of fostering genuine democratic deliberation, referendums are frequently exploited to secure partisan advantages, suppress political competition, and advance authoritarian agendas. The absence of institutional safeguards, such as parliamentary filters, exacerbates these risks by enabling majority rule to undermine minority rights and deepen societal divisions. These patterns highlight how direct democracy, when misused, can erode democratic principles and destabilize broader governance frameworks.

## Ethics and consent

Ethical approval and consent were not required.

## Data Availability

No data associated with this article.

## References

[ref-1] Ackermann K Braun D Fatke M : Direct democracy, political support and populism-attitudinal patterns in the German bundesländer. *Regional & Federal Studies.* 2023;33(2):139–62. 10.1080/13597566.2021.1919876

[ref-2] Acosta García N : Can direct democracy deliver an alternative to extractivism? An essay on popular consultations. *Polit Geogr.* 2022;98: 102715. 10.1016/j.polgeo.2022.102715

[ref-70] Adam EC Kagiaros D Tierney S : Democracy in question? Direct democracy in the European Union. *Eur Const Law Rev.* 2018;14(2):261–282. 10.1017/S1574019618000160

[ref-3] Adinolfi G : Direct democracy, ‘Plebiscitarianism’ and military rule: is the portuguese normative view of democracy multidimensional? *Port J Soc Sci.* 2018;17(2):229–48. 10.1386/pjss.17.2.229_1

[ref-4] Altman D : Direct democracy worldwide. Cambridge: Cambridge University Press,2011. 10.1017/CBO9780511933950

[ref-5] Altman D : The potential of direct democracy: a global measure (1900-2014). *Soc Indic Res.* 2017;133(3):1207–27. 10.1007/s11205-016-1408-0

[ref-6] Asimakopoulos J : A radical proposal for direct democracy in large societies. *Braz J Nat Sci.* 2016;36(2):430–47. 10.1590/0101-31572016v36n02a10

[ref-7] Barth J Burnett CM Parry J : Direct democracy, educative effects, and the (mis)measurement of ballot measure awareness. *Polit Behav.* 2020;42(4):1015–34. 10.1007/s11109-019-09529-w

[ref-8] Bermeo N : On democratic backsliding. *J Democr.* 2016;27(1):5–19. 10.1353/jod.2016.0012

[ref-9] Bernhard L : Does direct democracy increase civic virtues? A systematic literature review. *Front polit sci.* 2024;6. 10.3389/fpos.2024.1287330 1287330

[ref-10] But JJ Jongkind DK Voermans WJM : Direct democracy in the constitution: good or bad for democracy? *The Theory and Practice of Legislation.* 2023;11(1):52–82. 10.1080/20508840.2022.2131150

[ref-71] Chang WC : Direct Democracy and party membership: testing the role of political efficacy. *Polit Stud Rev.* 2023;21(1). 10.1177/14789299211034278

[ref-11] Childers M Binder M : Engaged by the initiative? How the use of citizen initiatives increases voter turnout. *Polit Res Q.* 2012;65(1):93–103. 10.1177/1065912910388191

[ref-12] Colombo C Kriesi H : Party, policy - or both? Partisan-biased processing of policy arguments in direct democracy. *J Elect Public Opin Parties.* 2017;27(3):235–53. 10.1080/17457289.2016.1254641

[ref-13] Cortés JJ : Process, theory, and practice in direct democracy: avenues for new research. *Politics Policy.* 2019;47(4):673–98. 10.1111/polp.12317

[ref-14] Dalton RJ Burklin WP Drummond A : Public opinion and direct democracy. *J Democr.* 2001;12(4):141–53. 10.1353/jod.2001.0066

[ref-15] Della Porta D : Can democracy be saved? Participation, deliberation and social movements. Cambridge: Polity,2013. Reference Source

[ref-16] Dvořák T Zouhar J Novák J : The effect of direct democracy on turnout: voter mobilization or participatory momentum? *Polit Res Q.* 2017;70(2):433–48. 10.1177/1065912917698043

[ref-17] Dyck JJ Seabrook NR : Mobilized by direct democracy: short-term versus long-term effects and the geography of turnout in ballot measure elections. *Soc Sci Q.* 2010;91(1):188–208. 10.1111/j.1540-6237.2010.00688.x

[ref-18] Economou EML Kyriazis NC Metaxas T : Ancient athenians, Californians and modern greeks: a comparative analysis of choice set under direct democracy procedures. *Homo Oeconomicus.* 2017;34(1):47–65. 10.1007/s41412-017-0039-2

[ref-63] el-Wakil A : The deliberative potential of facultative referendums: procedure and substance in direct democracy. *Democratic Theory.* 2017;4(1). 10.3167/dt.2017.040104

[ref-64] el-Wakil A McKay S : Disentangling referendums and direct democracy: a defence of the systemic approach to popular vote processes. *Representation.* 2020;56(4):449–66. 10.1080/00344893.2019.1652203

[ref-72] Freitag M Ackermann K : Direct democracy and institutional trust: relationships and differences across personality traits. *Polit Psychol.* 2016;37(5):707–723. 10.1111/pops.12293

[ref-19] Geißel B Krämling A Paulus L : More or less equality? Direct democracy in Europe from 1990 to 2015. *Z Vgl Polit.* 2019;13(4):491–525. 10.1007/s12286-019-00435-3

[ref-20] Gerber ER, ed : The populist paradox: interest group influence and the promise of direct legislation. Princeton: Princeton University Press,2011. Reference Source

[ref-21] Gerber M Mueller S : When the people speak – and decide: deliberation and direct democracy in the citizen assembly of Glarus, Switzerland. *Policy & Politics.* 2018;46(3):371–90. 10.1332/030557317X14976099453327

[ref-22] Gherghina S : Direct democracy and subjective regime legitimacy in Europe. *Democratization.* 2017;24(4):613–31. 10.1080/13510347.2016.1196355

[ref-23] Gherghina S : Hijacked direct democracy: the instrumental use of referendums in Romania. *East Eur Polit Soc.* 2019;33(3):778–97. 10.1177/0888325418800553

[ref-73] Gherghina S Pilet J : Populist attitudes and direct democracy: a questionable relationship. *Swiss Polit Sci Rev.* 2021;27(2):496–505. 10.1111/spsr.12451

[ref-24] Hainmueller J Hangartner D : Does direct democracy hurt immigrant minorities? Evidence from naturalization decisions in Switzerland. *Am J Pol Sci.* 2019;63(3):530–47. 10.1111/ajps.12433

[ref-25] Hajnal ZL Gerber ER Louch R : Minorities and direct legislation: evidence from California ballot proposition elections. *J Polit.* 2002;64(1):154–77. 10.1111/1468-2508.00122

[ref-26] Helfer L Wäspi F Varone F : Does direct democracy enhance politicians’ perceptions of constituents’ opinions? Evidence from Switzerland. *Swiss Political Science Review.* 2021;27(4):695–711. 10.1111/spsr.12495

[ref-27] Hibbing JR Theiss-Morse E : Stealth democracy: Americans’ beliefs about how government should work.Cambridge University Press,2002. 10.1017/CBO9780511613722

[ref-28] Inglehart R : Modernization and postmodernization: cultural, economic, and political change in 43 societies.Princeton University Press,1997. Reference Source

[ref-29] Kern A Hooghe M : The effect of direct democracy on the social stratification of political participation: inequality in democratic fatigue? *Comp Eur Polit.* 2018;16(4):724–44. 10.1057/s41295-017-0093-y

[ref-30] Kersting N Regalia M : Direct democracy and its integrity. The Italian 2020 and the Turkey 2017 constitutional referendums. *Z Vgl Polit Wiss.* 2022;16(3):527–44. 10.1007/s12286-022-00552-6

[ref-31] Kim JH : Direct democracy and women’s political engagement. *Am J Pol Sci.* 2019;63(3):594–610. 10.1111/ajps.12420

[ref-32] Kogan V Lavertu S Peskowitzt Z : Direct democracy and administrative disruption. *J Public Adm Res Theory.* 2017;27(3):381–99. 10.1093/jopart/mux001

[ref-33] Krämling A Geißel B Rinne JR : Direct democracy and equality: a global perspective. *Int Polit Sci Rev.* 2023;44(4):507–22. 10.1177/01925121211058660

[ref-34] Kriesi H : Direct democracy: the Swiss experience.In: *Evaluating Democratic Innovations.*Routledge,2012. Reference Source

[ref-35] Ladam C : Does process matter? Direct Democracy and citizens’ perceptions of laws. *Journal of Experimental Political Science.* 2020;7(3):232–37. 10.1017/XPS.2019.28

[ref-36] Ladner A Fiechter J : The influence of direct democracy on political interest, electoral turnout and other forms of citizens’ participation in Swiss municipalities. *Local Gov Stud.* 2012;38(4):437–59. 10.1080/03003930.2012.698242

[ref-37] Lascher EL Hagen MG Rochlin SA : Gun behind the door? Ballot initiatives, state policies and public opinion. *J Polit.* 1996;58(3):760–75. 10.2307/2960443

[ref-38] Lax JR Phillips JH : The Democratic Deficit in the states. *Am J Pol Sci.* 2012;56(1):148–166. 10.1111/j.1540-5907.2011.00537.x

[ref-39] Leemann L Wasserfallen F : The democratic effect of direct democracy. *Am Polit Sci Rev.* 2016;110(4):750–62. 10.1017/S0003055416000307

[ref-41] Matsusaka JG : For the many or the few the initiative, public policy, and American democracy. University of Chicago Press, 2004. Reference Source

[ref-42] Matsusaka JG : Popular control of public policy: a quantitative approach. *Quart J Polit Sci.* 2010;5(2):133–67. 10.1561/100.00009055

[ref-40] Matsusaka JG McCarty NM : Political resource allocation: benefits and costs of voter initiatives. *J Law Econ Organ.* 2001;17(2):413–48. 10.1093/jleo/17.2.413

[ref-43] McCoy J Rahman T Somer M : Polarization and the global crisis of democracy: common patterns, dynamics, and pernicious consequences for democratic polities. *Am Behav Sci.* 2018;62(1):16–42. 10.1177/0002764218759576

[ref-44] Mendelsohn M Cutler F : The effect of referendums on democratic citizens: information, politicization, efficacy and tolerance. *Br J Polit Sci.* 2000;30(4):669–98. Reference Source

[ref-45] Mendez F Mendez M : The promise and perils of direct democracy for the European Union. *Cambridge Yearbook of European Legal Studies.* 2017;19:48–85. 10.1017/cel.2017.7

[ref-46] Merli F : Illiberal direct democracy. *Vienna Online Journal on International Constitutional Law(ICL-Journal).* 2020;14(2):199–210. 10.1515/icl-2020-0001

[ref-48] Mudde C Rovira Kaltwasser C : Populismo. Uma Brevíssima Introdução. Lisboa: Gradiva,2017.

[ref-47] Mudde C : The populist zeitgeist. *Government and Opposition.* 2004;39(4):541–63. 10.1111/j.1477-7053.2004.00135.x

[ref-49] Orhan YE : The relationship between affective polarization and democratic backsliding: comparative evidence. *Democratization.* 2022;29(4):714–35. 10.1080/13510347.2021.2008912

[ref-50] Pállinger ZT : Direct democracy in an increasingly illiberal setting: the case of the Hungarian national referendum. *Contemporary Politics.* 2019;25(1):62–77. 10.1080/13569775.2018.1543924

[ref-51] Pantazi M Papaioannou K van Prooijen JW : Power to the people: the hidden link between support for direct democracy and belief in conspiracy theories. *Pol Psych.* 2022;43(3):529–48. 10.1111/pops.12779

[ref-52] Pauwels T : Populism in Western Europe: comparing Belgium, Germany and the Netherlands. 1st ed. Routledge,2014. 10.4324/9781315764313

[ref-53] Peters Y : Zero-sum democracy? The effects of direct democracy on representative participation. *Pol Sci.* 2016;64(3):593–613. 10.1177/0032321715607510

[ref-54] Prato C Strulovici B : The hidden cost of direct democracy: how ballot initiatives affect politicians’ selection and incentives. *J Polit Theory.* 2017;29(3):440–66. 10.1177/0951629816650762

[ref-55] Qvortrup M : The rise of referendums: demystifying direct democracy. *J Democr.* 2017;28(3):141–52. 10.1353/jod.2017.0052

[ref-56] Smith D Tolbert C : Educated by initiative: the effects of direct democracy on citizens and political organizations in the American States. Ann Arbor, MI: University of Michigan Press,2004. 10.3998/mpub.11467

[ref-57] Sunstein CR : The law of group polarization. *J Polit Philos.* 2002;10(2):175–95. 10.1111/1467-9760.00148

[ref-58] Tausanovitch C Warshaw C : Representation in municipal government. *Am Polit Sci Rev.* 2014;108(3):605–41. 10.1017/S0003055414000318

[ref-59] Tosun J Béland D Papadopoulos Y : The impact of direct democracy on policy change: insights from European citizens’ initiatives. 2022;323–340. 10.1332/030557321X16476244758073

[ref-60] Towfigh EV Goerg SJ Glöckner A : Do direct-democratic procedures lead to higher acceptance than political representation? *Public Choice.* 2016;167(1):47–65. 10.1007/s11127-016-0330-y

[ref-61] Van Outryve S : Realising direct democracy through representative democracy: from the Yellow Vests to a libertarian municipalist strategy in Commercy. *Urban Studies.* 2023;60(11):2214–30. 10.1177/00420980231156026

[ref-62] Vatter A Rousselot B Milic T : The input and output effects of direct democracy: a new research agenda. *Policy Politics.* 2019;47(1):169–86. 10.1332/030557318X15200933925423

[ref-65] Walter A : Taking the initiative: direct democracy, coalition governments and welfare state expansion. *J Eur Soc Policy.* 2019;29(3):446–59. 10.1177/0958928718805631

[ref-66] Wolkenstein F : What is democratic backsliding? *Constellations.* 2023;30(3):261–75. 10.1111/1467-8675.12627

