# Supplementary material for: Recent insights on direct democracy: Arguments, drivers, effects and conditions
Source: Open Res Eur. 2025 Jun 27;5:171. [Version 1] doi: 10.12688/openreseurope.20444.1 (PMC12368492; doi:10.12688/openreseurope.20444.1)
Supplement: Peer review history

# Reviewer report: Reviewer response for version 1

**DOI:** 10.21956/openreseurope.22123.r56478

**Published:** 2025-08-20

**Authors:** Hanno Scholtz

**Affiliations:** 1 University of Zurich, Zurich, Switzerland

The literature cut-off occurred more than a year and a half before the text reached me, which is a considerable gap in such a fast-moving field. At the same time, I appreciate the difficulty of writing a comprehensive overview in such a fertile research area. The authors’ decision to work the sample to its analytical limits—while resisting the temptation to apply statistical methods to what is essentially a qualitative thematic dataset—reflects a clear editorial choice, which I respect. Nevertheless, the result inevitably shows the limitations of that approach. 

 One main concern is with the sequence in which the six thematic portions of the literature are presented. In the current form, they are arranged more as a thematic collage than as a progression of insight. If the aim is to give readers a clear picture of what we know, it would be more effective to structure the synthesis along causal lines: begin with the drivers of direct democracy, then turn to design questions that can be discussed without reference to outcomes, then present the evidence on effects, and finally address studies of interaction effects with moderator variables. Such a structure would make the intellectual architecture of the field much more visible and would also better convey where the literature’s causal claims are strong and where they remain tentative.

 Today’s research fields are generally too large to cover comprehensively without some quantitative backbone. I see the study of direct democracy as necessarily interdisciplinary, drawing from political science, psychology, sociology, communication studies, and history. Over the past years, I have developed a practice of mapping mid-size literatures—between one and ten thousand items—through co-citation analysis, using principal components analysis on datasets with dummy variables for the 400 most-cited texts, 100 most-cited authors, and 30 most-cited journals. For comparison, I ran such an analysis here, deriving 12 debates discussed in 993 texts with “direct democra*” or “referend*” in the title plus adding 139 texts that ranked in the top 400 among 3,619 texts with those terms anywhere in the text, and restricting the search to the review’s time horizon (2016-01-01 to 2023-10-30).

 The result is a map of twelve distinct debates, which I will summarize here along the causal ordering I propose. In the “causes” category, Debate 12 on populism and issue voting finds the populism–direct democracy link to be more contingent than often assumed, with strategic support depending on issue and expected outcome (e.g., Gherghina & Pilet 2021; Werner & Jacobs 2022). Debate 2 on participation and equality spans causes and effects: participation gaps are shaped by socio-demographic inequalities, but such gaps also feed back into legitimacy perceptions (Kern & Hooghe 2018; Dvorák, Zouhar & Novák 2017).

 Under “design questions,” Debate 6 addresses institutional forms and procedural rules, comparing how different mechanisms operate across contexts (Gherghina & Silagadze 2021; Jäske 2017). Debate 10 looks at sovereignty and constitutional referendums, showing both the strategic calculus behind allowing such votes and their variable potential to resolve or inflame conflicts (Germann 2022; Harguindéguy, Sánchez & Sánchez 2021).

 The “effects” category contains several well-developed streams. Debate 1 on economic effects reports evidence for fiscal restraint and efficiency gains under certain conditions (Asatryan, Baskaran & Heinemann 2017; Emmenegger, Leemann & Walter 2022). Debate 4 on policy congruence finds that direct democracy can align policy with voter preferences, though often mediated by elite framing (Lewis & Jacobsmeier 2017; Farley 2021). Debate 5 on EU referendums examines their impact on integration politics and democratic legitimacy (Atikcan, Nadeau & Bélanger 2020; Silagadze & Gherghina 2018). Debate 9 on institutional trust and system support shows asymmetric winner–loser effects and the role of procedural fairness (Leemann & Stadelmann-Steffen 2022; Arnesen et al. 2019). Debate 11 on turnout and mobilization demonstrates that campaign intensity selectively boosts participation, while institutional rules such as quorums or compulsory voting enforcement can depress or stimulate it (Goldberg, Lanz & Sciarini 2019; Marx & Leininger 2022). Debate 8 on Brexit and national populism shows how identity, immigration attitudes, and economic grievances converge to drive referendum outcomes (Dennison, Davidov & Seddig 2020; Hobolt, Leeper & Tilley 2021).

 Finally, under “interaction effects,” Debate 3 on political support and legitimacy shows how prior trust levels and institutional quality condition direct democracy’s effects on legitimacy (Marien & Kern 2018; Werner, Marien & Felicetti 2020). Debate 7 on voter knowledge and deliberation demonstrates that information environments, partisan cues, and individual predispositions interact to shape competence and choice (De Angelis, Colombo & Morisi 2020; Colombo 2018).

 Comparing these insights with the grouping in the reviewed text shows partial overlap. Several themes—such as fiscal effects, participation patterns, legitimacy, and populism—are present in both. However, my analysis separates causes, design, effects, and interactions more sharply, while the reviewed text merges them thematically. Some areas from the larger map are missing in the review’s grouping, most notably: the economic-fiscal dimension (Debate 1) as a coherent theme; the distinct treatment of high-stakes sovereignty and constitutional referendums (Debate 10); and the systematic attention to information processing and heuristics in voter decision-making (Debate 7). The review also underrepresents interdisciplinary contributions from psychology (e.g. heuristics and bias research), communication science (campaign framing effects), and sociology (identity-driven participation patterns) which are important parts of the larger landscape.

 For a review that uses the traditional method of hermeneutically interpreting articles, the Şimşek et al. text does a fair job: the individual texts are well summarized, and important developments in the literature are pointed to. One can critique the choice of this traditional method, as its necessarily confined search strategy—most notably leaving out the large body of literature with “referendum” or “direct democratic” as keywords and omitting interdisciplinary insights outside of political science—leaves important findings unaddressed, such as the fiscal and budgetary impacts of direct democracy, the strategic logic behind sovereignty referendums, and the nuanced role of information environments in shaping voter competence. But of course, using a different methodology would have produced a fundamentally different text. My suggestion for the editors is to accept the text with reservations, and—if changes are still possible—to rearrange it using a causality-based structure, or at least to add a paragraph that discusses the relationship between the current thematic grouping and causal relations. For future reviews, I would encourage the authors to consider complementing their hermeneutic synthesis with quantitative mapping methods to better capture the breadth and interdisciplinary nature of this research field.

Are all factual statements correct and adequately supported by citations?

Yes

Is the review written in accessible language?

Yes

Are the conclusions drawn appropriate in the context of the current research literature?

Partly

Is the topic of the review discussed comprehensively in the context of the current literature?

Partly

Reviewer Expertise:

Political science and sociology, especially democracy models beyond traditional representative democracy

I confirm that I have read this submission and believe that I have an appropriate level of expertise to confirm that it is of an acceptable scientific standard, however I have significant reservations, as outlined above.

# Reviewer report: Reviewer response for version 1

**DOI:** 10.21956/openreseurope.22123.r56296

**Published:** 2025-08-05

**Authors:** Madeleine Hosli, Abdullah Shehata

**Affiliations:** 1 Leiden University, The Hague, The Netherlands

Anchored in four guiding questions and a clearly defined thematic scope, the article presents a well-organized and critically engaged synthesis of recent scholarship on direct democracy (albeit with some repetitions within the text). Drawing on 43 articles published between 2016 and 2023, the authors structure their discussion around key questions: what the drivers of the adoption of direct democracy are, under what conditions it succeeds, what its effects are, and how these dynamics unfold across political and institutional contexts. The review is explicitly framed as thematic rather than systematic, and the authors are transparent about its boundaries. Within those parameters, the article effectively captures the central debates and recurring concerns that shape the literature.

 The paper has many strengths. It draws on diverse empirical examples, ranging from citizen-driven referendums in Switzerland to elite-managed plebiscites in Hungary and Romania. It balances optimism about direct democracy’s participatory potential with clear-eyed attention to risks such as elite capture, populist instrumentalization, and the marginalization of minority groups. The article’s engagement with themes like political disillusionment, legitimacy crises, and democratic backsliding is particularly valuable. These issues are explored across both liberal and illiberal regimes and are especially relevant to scholarship in political science and international relations. Taken together, these contributions make the article a valuable resource for researchers seeking to understand the multiple and often contradictory roles of direct democratic instruments.

 That said, a few areas would benefit from further refinement. Although the authors justifiably limit their sample to articles with the term “direct democracy” in the title and clarify that the review is thematic rather than comprehensive, the implications of this selection strategy could be more explicitly acknowledged. This choice ensures coherence, but it also narrows the conceptual field and risks excluding studies that analyze referendums or participatory mechanisms under different terminologies. A brief reflection on how this might have shaped the thematic findings, as a trade-off of scope rather than an oversight, would strengthen the self-reflexive element of the review, particularly since the issue is mentioned in both the introduction and conclusion.

 Second, regarding the fourth guiding question on how political and institutional contexts shape the dynamics of direct democracy, the article presents a wide range of national case studies, but their treatment remains largely illustrative. Examples from Hungary, Switzerland, Colombia, and Turkey are presented individually rather than synthesized around shared conditions or patterns. While the authors explicitly refrain from offering a systematic comparison, there is still room to organize contextual insights more clearly. Applying a comparative lens by grouping findings according to regime type, legal constraints, or administrative capacity could help clarify the conditions under which specific risks or benefits tend to emerge. To remain consistent with the authors’ stated aims, this suggestion pertains to the structuring of insights already present in the literature, rather than the introduction of original empirical claims. It would also help to define what the authors consider to be elements of direct democracy (next to referendums or initiatives), including the extent to which citizens are able to make suggestions for constitutional amendments -- based on voter signature requirements -- or to initiate referendums on laws issued by the national parliament.

 Third, the second guiding question, which addresses the conditions under which direct democracy succeeds, is discussed across several sections but without a clear definition of success. The review references enabling factors such as civic culture, institutional safeguards, or deliberative quality. However, it is not always clear whether “success” refers to legitimacy, participation, congruence between policy and public preferences, or regime responsiveness. A more explicit conceptual framework, such as one that distinguishes between input, throughput, and output legitimacy, would clarify how different studies understand and evaluate success and would also improve the coherence of the synthesis.

 Fourth, while the review highlights important normative themes such as legitimacy, populism, and democratic disillusionment, these are introduced primarily as topical categories rather than analytically developed frameworks. In many cases, the review summarizes how individual studies refer to these issues without explaining how they are defined, contested, or connected across the literature. This limits the article’s ability to clarify the conceptual stakes of the patterns it identifies. A more explicit effort to connect these themes to broader theoretical debates already present in the reviewed scholarship could help readers to better understand both the assumptions underlying different studies and the significance of their findings for the study of democratic governance.

 Finally, there are minor editorial issues, such as inconsistent spelling of names (e.g., “Leeman” vs. “Leemann”) and occasional typographic slips (e.g., doubled parentheses). These are superficial and easily corrected, but worth addressing. The reference Walter (2019) is mentioned in the main text but not listed in the bibliography.

 In sum, this is a well-written and timely review that draws together diverse case studies, benefits and risks of direct democracy in the scholarship with clarity and critical insight. Our suggestions are intended to sharpen rather than restructure the article. By clarifying the implications of its scope, avoiding repetition, offering a more structured comparative lens, and deepening the conceptual framework, the review can become even more effective in guiding both scholarly debate and future research on the evolving role of direct democracy in contemporary governance.

Are all factual statements correct and adequately supported by citations?

Yes

Is the review written in accessible language?

Yes

Are the conclusions drawn appropriate in the context of the current research literature?

Yes

Is the topic of the review discussed comprehensively in the context of the current literature?

Yes

Reviewer Expertise:

International Political Economy, International Organization, European Integration.

We confirm that we have read this submission and believe that we have an appropriate level of expertise to confirm that it is of an acceptable scientific standard.

# Reviewer report: Reviewer response for version 1

**DOI:** 10.21956/openreseurope.22123.r56297

**Published:** 2025-07-21

**Authors:** Thom Oliver

**Affiliations:** 1 University of the West of England, Bristol, UK

**Summary of the article**

 This article provides a structured thematic review of English-language scholarship on direct democracy published between 2016 and 2023. Based on a sample of 43 peer-reviewed articles, it maps recent developments in four interrelated areas: (1) arguments in favour of direct democracy, (2) drivers of its adoption, (3) empirical and perceived effects, and (4) enabling or constraining conditions for its operation. The authors do not aim to assess direct democracy’s performance per se, but to offer a synthesis of contemporary scholarly debates, highlighting tensions, risks, and normative expectations associated with direct democratic instruments.

 The article is framed explicitly as a review of existing scholarship, not as an intervention grounded in primary empirical evidence or normative political theory. Its ambition is to consolidate a literature that has grown in volume and complexity over the past decade, and to identify where recent findings converge, where they diverge, and where they raise new questions about the uses, limitations, and consequences of direct democratic practice. In doing so, it seeks to provide a resource for scholars interested in democratic innovation, legitimacy, and institutional design across a range of political systems.

 I have scored the article using the criteria above, and I add comments below, to explain those individual evaluations.

**Is the topic of the review discussed comprehensively in the context of the current literature?**

 This was scored as **partly. **The article and its authors succeed in delivering what is a well structured and reasonably broad survey of the recent literature. It is organised well thematically, and is presented clearly. It is able to capture the main strands of debate, including the emergent tensions between legitimacy and manipulation, and the 'ambivalent' relationship between direct democracy and population, and the critical tension regarding the educative or egalitarian potential of direct democracy initiatives.

 There are a handful of absences which limit the comprehensiveness of the review, even within its declared scope.

 Firstly, conceptual or typological precision could be stronger. For example the article refers to 'direct democracy' broadly, but does not consistently differentiate between referendums, initiatives, citizens assemblies, or other institutional or organisational forms. Not does it systematically classify cases by design, the much contested aspects of direct democracy in practice (binding versus advisory, top down versus bottom up), considerations such as procedural rules (turnout rules, agenda setting power), or normative processes (veto, consultation, or a broad consideration of empowerment).

 Whilst this may not be the defined scope of the review, the choice not to define this limitation or caveat it, tends to lead to a flattening of important institutional variation, which itself has implications for the outcomes later discussed.

 There is also a lack of consideration of a number of highly relevant subfields, or at least they are under-represented across the review, perhaps due to the inclusion/exclusion criteria) for example literature on digital forms of direct democracy. participatory platforms, or the grey areas of hybrid models of direct democracy are not considered. I would have anticipated to see works represented here from Helene Landemore, Graham Smith, and James Fishkin, who all published across the review period around relevant 'effects' which have reshaped the terrain, around deliberative quality, legitimacy, which might be broadening the reviews planned analytical scope, but probably worth another consideration, to rule in or rule out. It would certainly enrich analytical scope and add value.

 Theoretical depth is a little limited, whilst the article does an excellent job of mapping empirical finding and summarising arguments, it stops short of offering a synthetic classification, or critical framework. Concepts such as trust, legitimacy and congruence are invoked rather than problematised. Whilst the review clearly aims not to deliver a normative intervention, it may be worth a consideration, or more likely a critical stance, on how these concepts are deployed (non-uniformly, or uniformly across the cases that are reviewed and cited.

 Therefore these critical dimensions in particular, noting that broadening normatively is out of scope as presently dratted, perhaps constrain the articles capacity to surface deeper patterns, or guide future scholarship beyond emuneration. The review is informative and well organised, but a more critical synthesis, or theoretical integration would strengthen its analytical contribution  to the field significantly.

**2. Are all factual statements correct and adequately supported by citations? YES**

 The article is carefully referenced and evidentially sound. All claims are supported with relevant citations, often triangulated with multiple sources. The authors accurately represent the findings of the reviewed studies, distinguishing where appropriate between consensus and disagreement.

 There is commendable attention to nuance across diverse empirical contexts, and the authors avoid overgeneralisation. Studies from Switzerland, Romania, Greece, and Latin America are accurately characterised, and methodological heterogeneity is acknowledged in places. The inclusion of both quantitative and qualitative sources, as well as theoretical essays and institutional case studies, adds depth and diversity to the synthesis.

 There is room for improved clarity in a few areas for instance, where empirical effects are summarised in general terms, more specificity (e.g. regarding sample size, scope, or methodological variation) could aid interpretation but these are minor refinements rather than substantive concerns and may sit more widely as part of an alternative systematic literature review. Future iterations could also consider discussing variation in research design quality (e.g. cross-national panels vs. single-case ethnographies) to further strengthen the evidential base.

**3. Is the review written in accessible language?**

 The article is written in clear, accessible prose. Technical terminology is used sparingly and appropriately. The thematic structure and consistent signposting support reader navigation. The inclusion of a plain language summary is a valuable feature and well-executed. This is a particularly welcome element for a journal aiming to encourage broad engagement and policy-facing dialogue, and the authors have succeeded in making complex arguments digestible to a non-specialist audience without sacrificing substance.

 A small number of paragraphs are overly dense (especially in the central sections), and the inclusion of summary tables or visual aids could enhance readability. The addition of a table summarising the reviewed studies by country, method, institutional type, or key finding would significantly aid comprehension and provide a helpful reference point for future researchers.

 Nevertheless, the article remains accessible to an interdisciplinary audience. In this regard, the authors strike an effective balance between scholarly rigour and communicative clarity.

**4. Are the conclusions drawn appropriate in the context of the current research literature?**

 This was scored as **partly. **The conclusions are appropriately cautious and consistent with the review’s stated scope. The authors clearly refrain from offering normative or empirical generalisations about the effectiveness of direct democracy. Instead, they present a qualified synthesis: direct democracy holds democratic promise under certain institutional and cultural conditions but also carries significant risks, particularly in illiberal or highly polarised contexts.

 This framing is appropriate given the diversity of findings reviewed, and the authors are careful to avoid overstating the coherence or consensus within the field. The concluding caution about elite capture, democratic backsliding, and institutional fragility is particularly timely and well-supported.

 However, the conclusions remain largely descriptive. The article does not interrogate unresolved tensions in the field, such as the paradox between participation and overload, or the simultaneous appeal of direct democracy to both populist and deliberative ideals. The authors stop short of offering a normative interpretation of the literature or of engaging the theoretical stakes of the tensions they identify.

 Nor does the article offer an explicit research agenda or typology of outstanding questions. This is a missed opportunity. Given the breadth of literature reviewed, the authors are well-positioned to identify the next frontiers for empirical research (e.g. comparative design effects, democratic innovations beyond the West), methodological refinement (e.g. measuring congruence or legitimacy), or theoretical integration (e.g. bridging participatory and epistemic accounts).

 In short, while the conclusions are appropriate, they are not especially analytical. A stronger final section synthesising the implications of recent scholarship, identifying fault lines or gaps, and proposing directions for future work would raise the article’s overall contribution.

**Some Broader Comments**

**Analytical Framework**

 The review would benefit from a more clearly defined conceptual apparatus for categorising and interpreting the literature. The organising themes of drivers, effects and conditions, are helpful descriptively, but are not treated analytically, as they vary across institutional, normative (out of scope) and epistemic lines.

**Clarity of review design**

 The authors acknowledge this is not a systematic review, however the methodology used in the article selection, filtering, and synthesis, lacks a little transparency, for a high quality narrative review.

 The decision to include only articles with 'direct democracy' in the title, is perhaps overly narrow, and risks omitted highly relevant studies that address referendums, citizens initiatives and mini publics, as well as alternative technologies/digital innovations. A broadening of scope though effort and adding complexity would raise the contribution of the review significantly. 

 The authors describe the combination of google scholar and Scopus, without a clear justification of inclusion/exclusion protocols, and therefore this may introduce concerns about replicability and the presence/weight of grey literature. The review doesn't seem to clarify whether editorial filtering or methodological quality were applied.

 Therefore put broadly, the four main recommendations to try to address would be to 

*Strengthen the analytical framework,* which would elevate the review from a summary, to a more critical synthesis, and allow the authors to surface deeper patterns.

*Improve description of methodological transparency,* as noted above, clearly state criteria for inclusion, and scope, perhaps use tables to summarise the articles by geography, method, democratic instrument, and critically reflect on the reviews limitations.

*Engage more broadly/widen scope.* If the authors opted to widen search criteria, it would make a more significant contribution, or signpost towards literatures to enrich future work.

*Shift beyond synthesis, *there is an opportunity to shift beyond synthesis to identify tensions, contradictions, and conceptual gaps in the literature. This may well be a future, additional step for the author team, but for this review, a signpost towards an emerging research agenda or conceptual roadmap, would deliver a much needed forward looking orientation for a vital, and fast moving contested democratic domain.

Are all factual statements correct and adequately supported by citations?

Yes

Is the review written in accessible language?

Yes

Are the conclusions drawn appropriate in the context of the current research literature?

Partly

Is the topic of the review discussed comprehensively in the context of the current literature?

Partly

Reviewer Expertise:

I have published in the domains of democratic governance, and studied referendums as part of my work with the Qualitative Election Study of Britain.  From a process and methodological perspective, I have expertise in publishing systematic literature reviews.

I confirm that I have read this submission and believe that I have an appropriate level of expertise to confirm that it is of an acceptable scientific standard, however I have significant reservations, as outlined above.
